# Behavioral and Metabolic Effects of the Atypical Antipsychotic Ziprasidone on the Nematode *Caenorhabditis elegans*

**DOI:** 10.1371/journal.pone.0074780

**Published:** 2013-09-19

**Authors:** Priscila Gubert, Gabriel Costa Aguiar, Tácito Mourão, Jessika Cristina Bridi, Alexandre Guimarães Barros, Félix Alexandre Soares, Marco Aurélio Romano-Silva

**Affiliations:** 1 Departamento de Saúde Mental, Faculdade de Medicina, Universidade Federal de Minas Gerais, Belo Horizonte, Brazil; 2 Departamento de Química, Centro de Ciências Naturais e Exatas, Universidade Federal de Santa Maria, Santa Maria, RS, Brazil; 3 INCT de Medicina Molecular, Faculdade de Medicina, Universidade Federal de Minas Gerais, Belo Horizonte, Brazil; Kyushu University, Japan

## Abstract

Atypical antipsychotics are associated with metabolic syndrome, primarily associated with weight gain. The effects of Ziprasidone, an atypical antipsychotic, on metabolic syndrome has yet to be evaluated. Here in, we evaluated lipid accumulation and behavioral changes in a new experimental model, the nematode *Caenorhabditis elegans* (*C. elegans*). Behavioral parameters in the worms were evaluated 24 h after Ziprasidone treatment. Subsequently, lipid accumulation was examined using Nile red, LipidTox green and BODIPY labeling. Ziprasidone at 40 µM for 24 h effectively decreased the fluorescence labeling of all markers in intestinal cells of *C. elegans* compared to control (0.16% dimethyl sulfoxide). Ziprasidone did not alter behaviors related to energetic balance, such as pharynx pumping, defecation cycles and movement. There was, however, a reduction in egg-production, egg-laying and body-length in nematodes exposed to Ziprasidone without any changes in the progression of larval stages. The serotoninergic pathway did not appear to modulate Ziprasidone’s effects on Nile red fluorescence. Additionally, Ziprasidone did not alter lipid accumulation in *daf-16* or *crh-1* deletion mutants (orthologous of the transcription factors DAF-16 and CREB, respectively). These results suggest that Ziprasidone alters reproductive behavior, morphology and lipid reserves in the intestinal cells of *C. elegans*. Our results highlight that the DAF-16 and CREB transcription factors are essential for Ziprasidone-induced fat store reduction.

## Introduction

Antipsychotics are used for treatment of mental illness [1]. Among them the newer atypical antipsychotics (AAP) have been ascribed to have superior therapeutic efficacy compared to the older typical antipsychotics (CATIE). Nevertheless, adverse effects mainly related to metabolic disruption are a major drawback associated with their use [2]. For instance, patients treated with Clozapine have increased risk for developing impaired glucose metabolism, weight gain and dyslipidemia [3,4] likely as a result of their interactions with the monoaminergic system. Moreover, there is remarkable heterogeneity risk for metabolic imbalances amongst different AAPs [3]. Clinical studies suggest that the AAP Ziprasidone does not cause several of those metabolic side effects induced by AAP drugs, primarily weight gain. Furthermore, it has been shown that Ziprasidone may be an effective adjuvant in weight loss therapy for overweight bipolar patients [5]. However, the pharmacological reasons for it are unclear. Data from positron emission tomography studies indicate that Ziprasidone has a high 5-HT2/D2 receptor occupancy fraction [6]. Also, it activates the 5-HT1a receptors and inhibits serotonin and norepinephrine uptake. Nevertheless, the relationship between pharmacodynamic features and metabolic outcomes of Ziprasidone are difficult to explore within a clinical context.

*Caenorhabditis elegans* is a free-living nematode that is well-established as a model for study of fat metabolism. Many aspects of energy related pathways are conserved between worms and mammals. This includes a regulatory role for neurons [7]. Through a simple neuronal circuitry, worms gauge the environment and internal sources of energy to modulate metabolism and foraging behaviors. Studies have pointed to serotonin, dopamine and other biogenic amines as key components of the system [8,9]. For example, worms with loss-of-function mutation in the serotonin reuptake pump show decreased fat depots [10].

Here we use the nematode *C. elegans* to explore the metabolic effects of Ziprasidone, aiming to gain insight into the molecular pathways its influence on lipid accumulation.

## Materials and Methods

### Culture conditions and strains

Worms were grown and maintained under standard conditions using *Escherichia coli* OP 50 as a food source as described previously [11]. The wild type reference strain was N2 Bristol. Additional strains included: *ser-4*(*ok512*) *III*, *ser-1*(*ok345*) *III*, *ser-7*(*tm1325*) *X*, *ser-3*(*ok1995*) *I*, *mod-1*(*ok103*) *V*, *mod-5*(*n822*) *I*, *tph-1*(*mg280*) *II*, *daf-16*(*mu86*) *I*, *ser-6*, *crh-1*(*tz2*) *III*. These strains were kindly provided by the CGC, except for the s*er-6* mutant, which was a gift from the Kenyon Lab. For each experiment synchronized populations were obtained through bleach treatments of gravid adults. Worms were used at the desired larval stage, as indicated below.

### Ziprasidone and monoamines treatment conditions

Ziprasidone (Sigma Aldrich) in DMSO (dimethyl sulfoxide) was freshly diluted in acetic acid (1:10.000) and added to the top of NGM (nematode growth medium) plates seeded with bacteria to the desired concentration. Octopamine (Sigma), dopamine (Sigma), serotonin (Sigma) and tyramine (Sigma) were dissolved in 0.1 N HCl and added to seeded NGM plates to the desired concentration. Each experimental condition was run in duplicate with appropriate controls and repeated at least twice.

### Nile Red, BODIPY and LipidTox Green labeling

Nile Red and C1-BODIPY-C12 conjugated fatty acids (BODIPY) lipid staining were carried out as previously described [12,13]. Dye stock solutions were diluted and added to NGM plates seeded with bacteria at final concentrations of 50 nM (Nile Red) and 0.05 µg/mL (BODIPY). Animals were grown and treated on plates containing Nile Red or BODIPY. Worms were mounted on agar pads and immobilized with sodium azide for image acquisition. Images were acquired using identical settings and appropriate filters with a Zeiss Axiovert II microscope fitted with a CCD camera. The time response curve on lipid accumulation was verified after Ziprasidone treatment through Nile Red labeling follow the protocol. The pictures were acquired at the same larval stage and the treatment started in different times before adult larval stage. LipidTox Green (Invitrogen) staining was carried out as previous described [14]. Briefly, worms were fixed in 2% paraformaldehyde, permeabilized with DTT and exposed to the staining solution containing 1:1000 LipidTox Green overnight. Images were acquired with a Leica LSCM system.

Image analyzes were conducted with ImageJ software. Fluorescence mean of the first two pairs of intestinal cells were used for analyzes.

### Pharyngeal pumping

Feeding rates were measured following previous protocol [15]. Briefly, pharyngeal bulb contractions were measured at room temperature in healthy adults on food for each condition. The number of contractions was counted over 10s in triplicate and the average used for statistical analyses. For each condition, ten animals were used. Each experiment was repeated at least twice.

### Defecation behavior

Defecation assays were performed at 22 °C ± 2 °C as previously described [15]. Worms on food were monitored and the interval between defecation cycles recorded. The mean of three defecation cycles from each animal was used as an indirect measurement of intestinal traffic. Ten worms were used per experiment. Experiments were repeated at least twice.

### Development and body length measurement

Worms were monitored from L1 larval stage until 72 h post adult molt. Body morphology was used to classify them. The perimeter was calculated using NIS-Element AR 3 software (Nikon).

### Egg laying and egg-production

Gravid adults were transferred to NGM plates and let lay eggs for 2 hours [15,16]. The next day, the number of progeny was counted as a measurement of eggs laid per worm. To assess the number of eggs inside the uterus, worms were burst and released eggs counted. Experiments were performed at room temperature and repeated at least twice.

### Locomotion assays

Worms were individually transferred to assay plates and 1 minute video was recorded with a stereomicroscope (Nikon – SMZ 1500) fitted with a CCD camera at a 12 frames/second. Speed, acceleration and distance were determined using the analysis software NIS-Elements AR 3 (Nikon). The experiments were carried out at 22 °C and repeated at least twice.

### Statistical analysis

Statistical analysis was performed using *GraphPad* Instat (Version 5.0 for Macintosh OSX, GraphPad Software, San Diego, CA). Significant differences between means were assessed by one-way ANOVA followed by Bonferroni’s post-hoc test.

## Results

### Ziprasidone reduces fat depots in *C. elegans*

**Figure 1:**
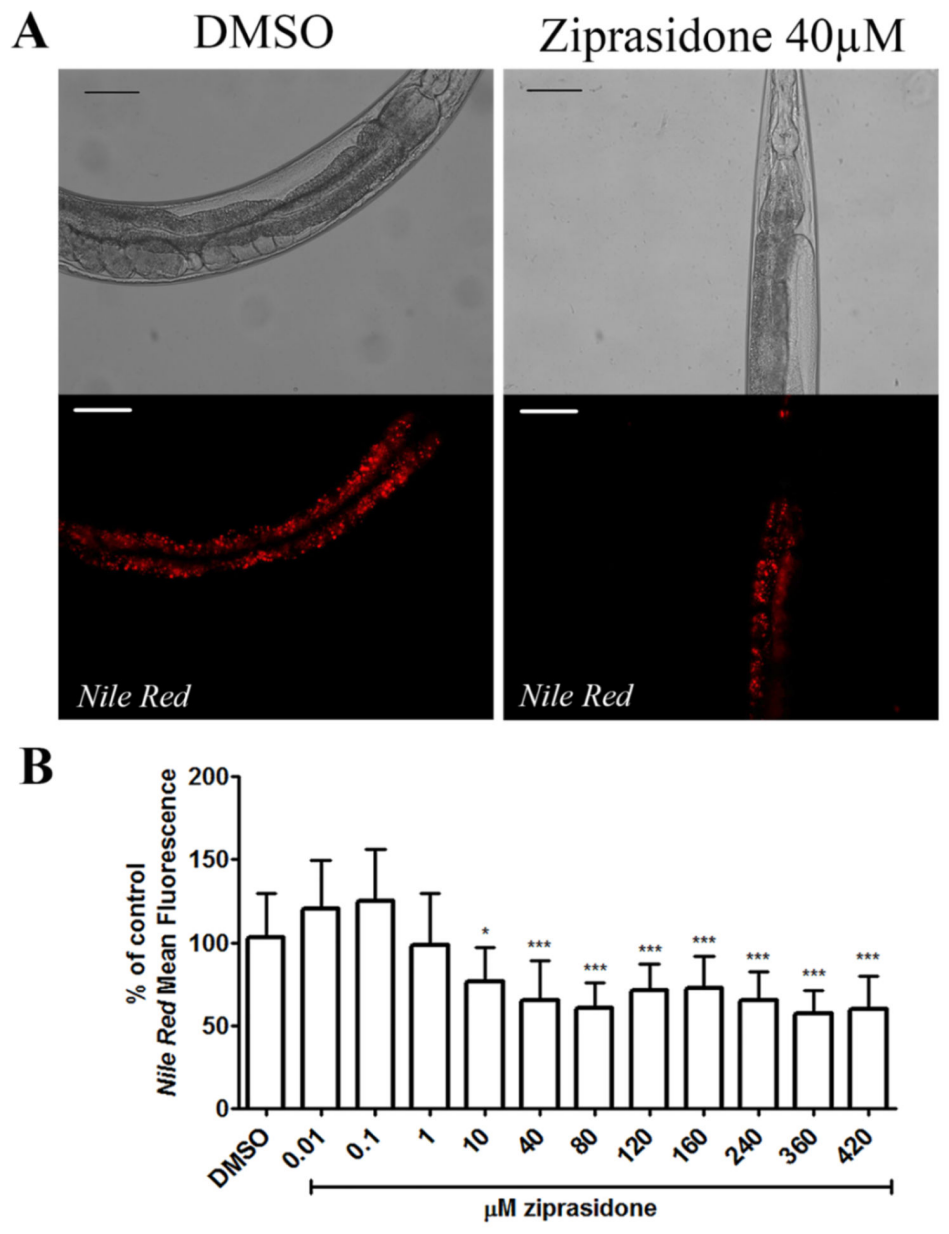
*Nile* *Red* Fluorescence reduction in *Caenorhabditis elegans* wild-type (N2) after Ziprasidone treatment for 24h. (A) Representative images of Ziprasidone treatment; (B) dose-response curve to Ziprasidone. A set of optical sections of 2.52 mm through the specimen (called a "Z series") of the first intestinal pair cells. The control group correspond to DMSO 0.16% in Acetic acid (1:10 000). The results were expressed in percentage of control (mean of fluorescence, standard deviation (SD), n>15). * p<0.05; ***p<0.0001 statistically different when compared to control 24h by one way ANOVA with Bonferroni correction for post hoc multiple comparisons.

**Figure 2:**
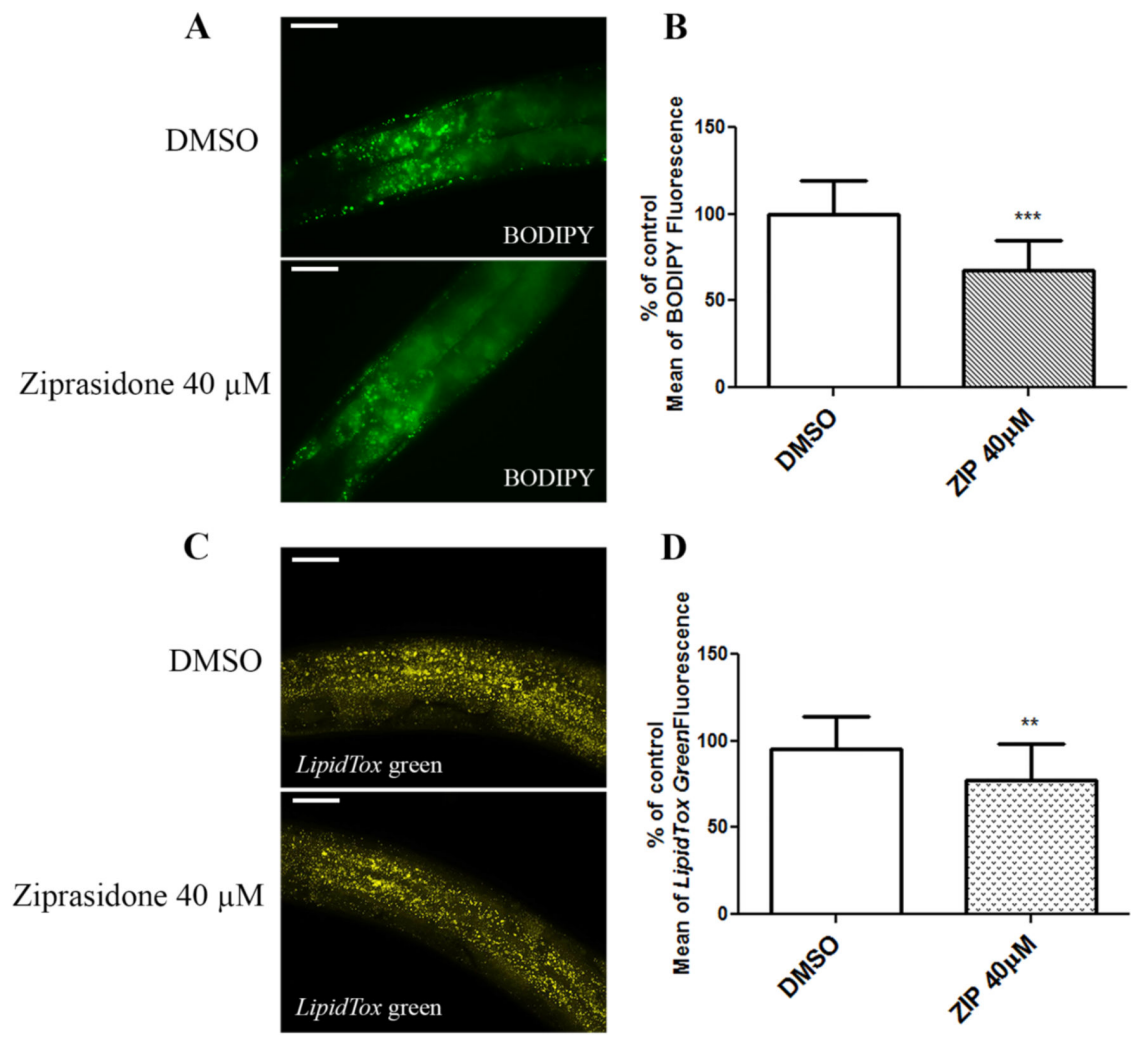
Fluorescence decrease induced by Ziprasidone exposition in *Caenorhabditis elegans* wild-type (N2) for 24 h. (A) Representative images of fat stores evidenced by BODIPY labeling and (B) fluorescence quantification; (C) Illustrative images and (D) densitometric quantification follow the fluorophore LipidTox Green labeling. The treatment with Ziprasidone (40 µM) or control (DMSO 0.16% in Acetic acid (1:10 000)) began in L4 larval stage until adult for 24 h. **p<0.01; ***p<0.0001, statistically different compared to the control 24h by one way ANOVA with Bonferroni correction for post hoc multiple comparisons, mean, SD, n= 10-25). The drug was poured and precipitated on agar plates and experiment performed two times at different days.

**Figure 3:**
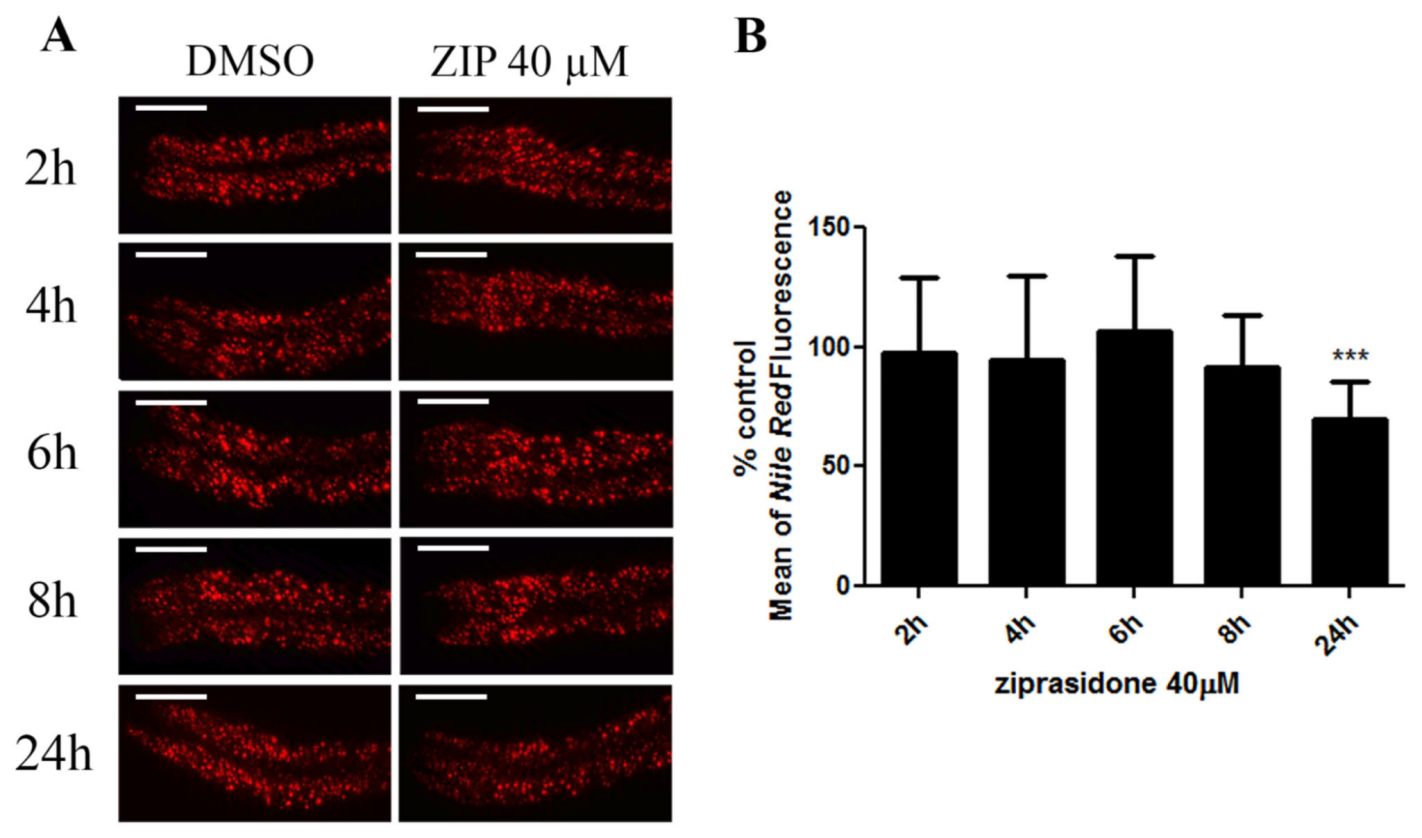
Ziprasidone activity-time curve on *Nile red* fluorescence in *Caenorhabditis elegans*. (A) *Nile* *Red* fluorescence in wild-type adult worms (N2) after Ziprasidone (40 µM) treatment; (B) Activity-time curve for 2, 4, 6, 8 and 24 hours. At the image acquisition time, all worms were in the same larval stage (adult). Data were normalized in percentage of each time respective control. Just in 24h hours, Ziprasidone significantly reduced the *Nile* *Red* fluorescence (***p<0.001, by ANOVA with Bonferroni correction for *post* *hoc* multiple comparisons, mean, SD, n>20-30).

Ziprasidone is associated with lesser metabolic side effects compared to other AAPs. To evaluate whether Ziprasidone modifies fat metabolism, worms were exposed over a concentration range, and their fat depots were assessed through vital dye fat staining. We observed a dose-dependent reduction in fluorescence in worms exposed to Ziprasidone (Figure 1). The results were confirmed by staining with two other dyes, BODIPY (Figure 2A and 2B) and LipidTox (Figure 2C and 2D). The decrease in fluorescence was only observed after 24 h Ziprasidone treatment (Figure 3). Combined, these data suggest that exposure to Ziprasidone decreases fat stores.

### Ziprasidone effects on *C. elegans* behaviors

**Figure 4:**
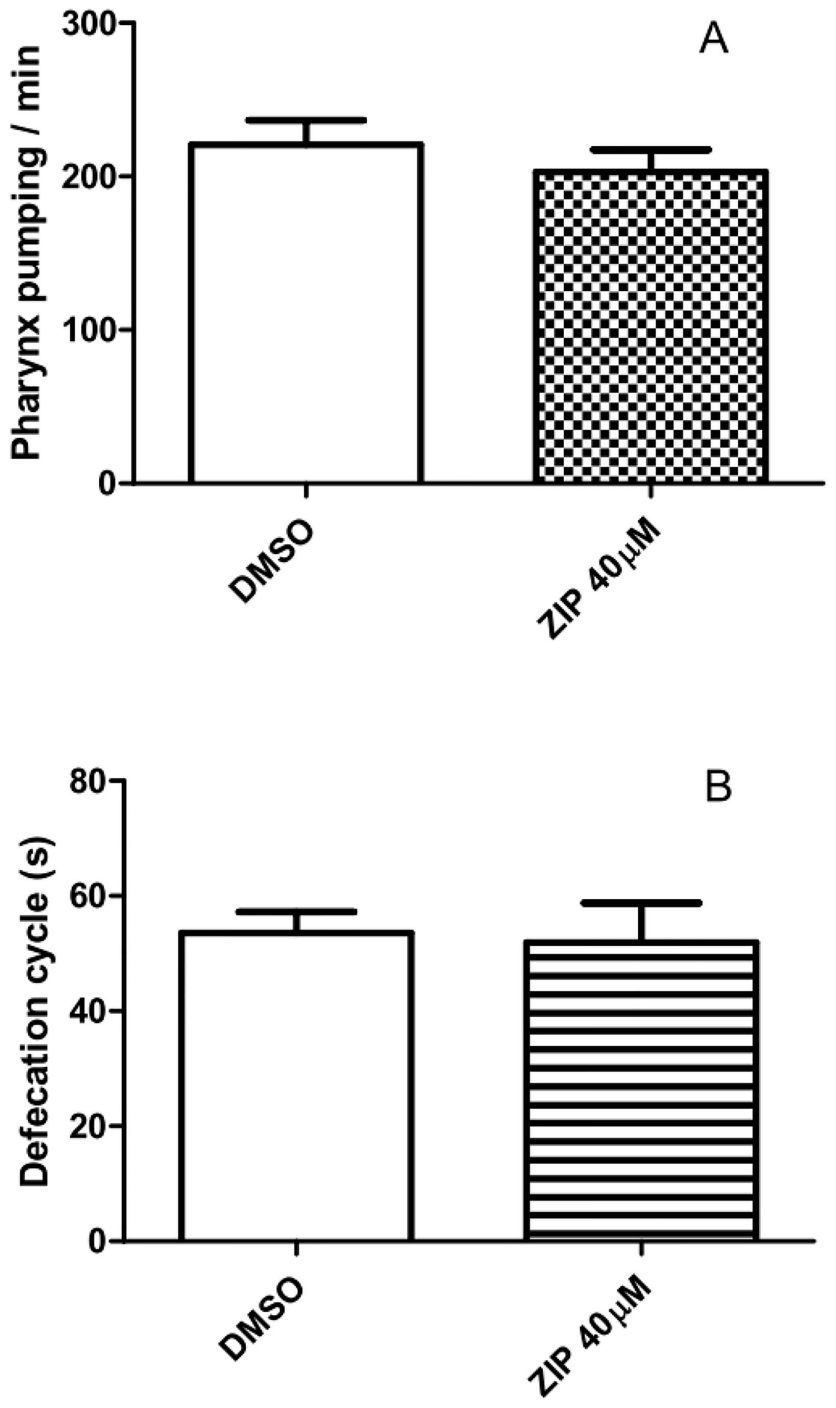
*Caenorhabditis elegans* wild-type behavior after Ziprasidone treatment for 24h. The worms were observed as the pharynx pumping/ min (A) and intervals of defecation cycles (s) (B) on treatment plates. The treatment with Ziprasidone (40 µM) or control (DMSO 0.16% in Acetic acid (1:10 000)) started in L4 larval stage for 24 h (adult). No statistic difference was found compared to control (0.16% DMSO) (mean, SD, n=20). The drug was poured and precipitated on agar plates.

Worms’ fat storage mirrors the balance between energy intake and consumption. Therefore it is possible that the decrease in fat stores induced by Ziprasidone originates from reduction in food intake. To evaluate whether Ziprasidone modifies feeding behavior, we analyzed the pharyngeal pumping rate and defecation cycle intervals. Both parameters were similar to controls in the presence of Ziprasidone (Figure 4). Thus, it a decrease in food intake does not appear to account for the observed fat reduction phenotype in response to Ziprasidone treatment.

**Figure 5:**
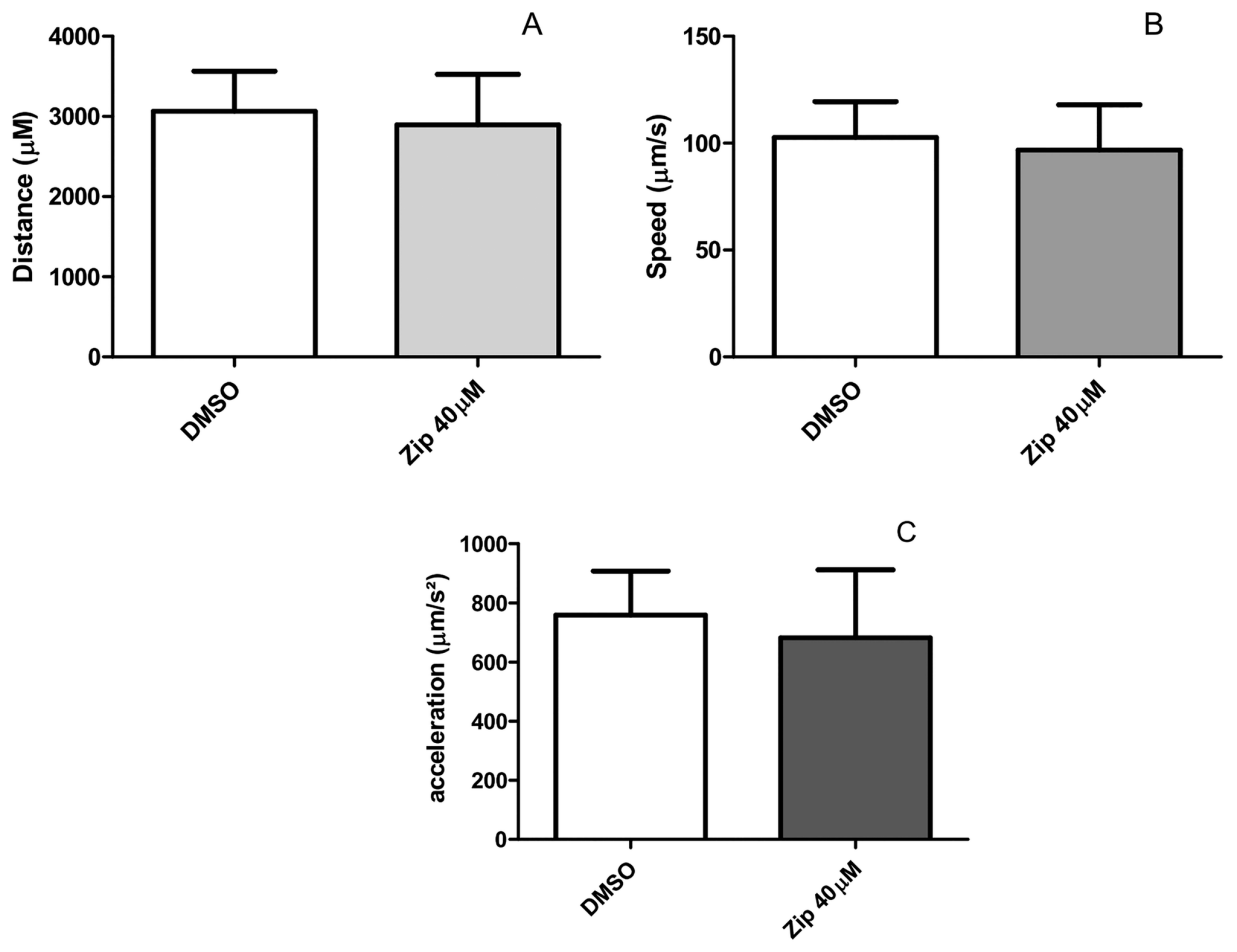
*C. elegans* wild-type movement behavior after Ziprasidone treatment for 24h. (A) Distance (µm), (B) speed (µm/s) and (C) acceleration (µm/s^2^) were quantified during 30 s after 24 h of Ziprasidone (40 µM) or vehicle exposition (DMSO 0.16% in Acetic acid (1:10 000)) started in L4 larval stage for 24 h (adult). No statistic difference was found compared to control (0.16% DMSO), mean, SD, n>6. The experiment was repeated three times in distinct days.

Movement parameters, such as distance traveled, speed and acceleration were unchanged compared to the control worms after Ziprasidone treatment (Figure 5). These findings indicate that the lipid storage phenotype induced by Ziprasidone treatment was not caused by an increase in body movements.

**Figure 6:**
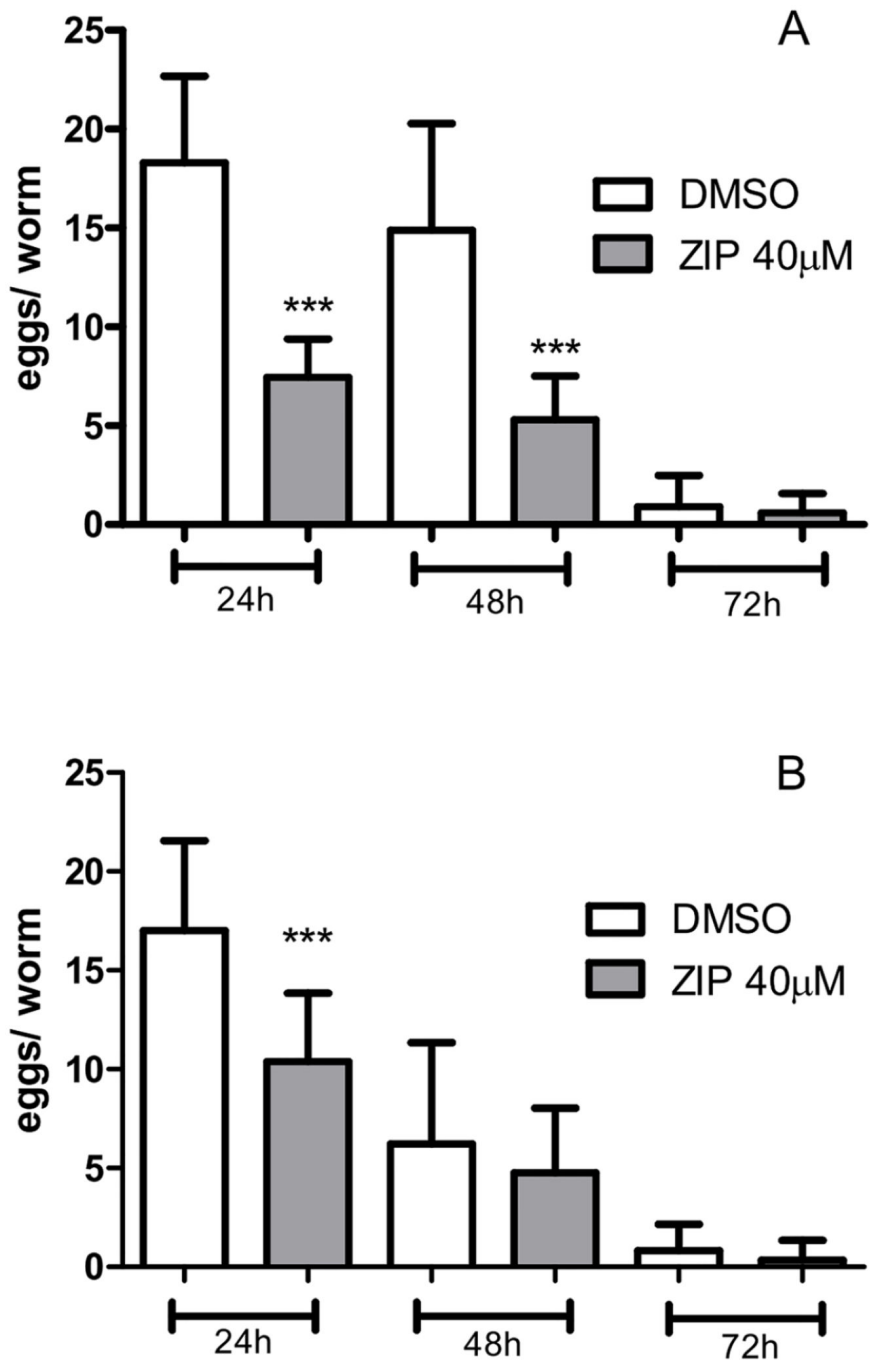
Reproductive behavior of the nematode *C. elegans* under Ziprasidone exposition. (A) The egg production and (B) egg laying were observed after 24, 48 and 72 hours of Ziprasidone treatment (40 µM) or vehicle as control (DMSO 0.16% in Acetic acid (1:10 000)) (mean, SD, n> 20)). The results represent the mean of eggs inside the worm (A) and the mean of the eggs released during 2 h in different time of treatment (24, 48 and 72 hours). ***p<0.0001, statistically different when compared to respective control of each exposition time by one way ANOVA with Bonferroni correction for *post* *hoc* multiple comparisons.

Worms spend part of their energy on reproduction. Embryos use yolk transferred from the intestine to the gonadal arms as a primary food source [17]. Therefore, we posited that Ziprasidone may reduce neutral lipid stores indirectly through modifications of the reproductive behavior. To elucidate the effects of Ziprasidone on reproduction, we counted the number of eggs produced after treatment. Curiously, Ziprasidone reduced the number of eggs inside the uterus (Figure 6A). The quantity of eggs laid overtime (Figure 6B), however, was lower only in the first 24 hours after Ziprasidone treatment. Accordingly, reproduction upon Ziprasidone treatment is correlated with energy accumulation. At this point, we are unable to exclude the possibility that disruption in egg production is an indirect consequence of fat store depletion by Ziprasidone.

**Figure 7:**
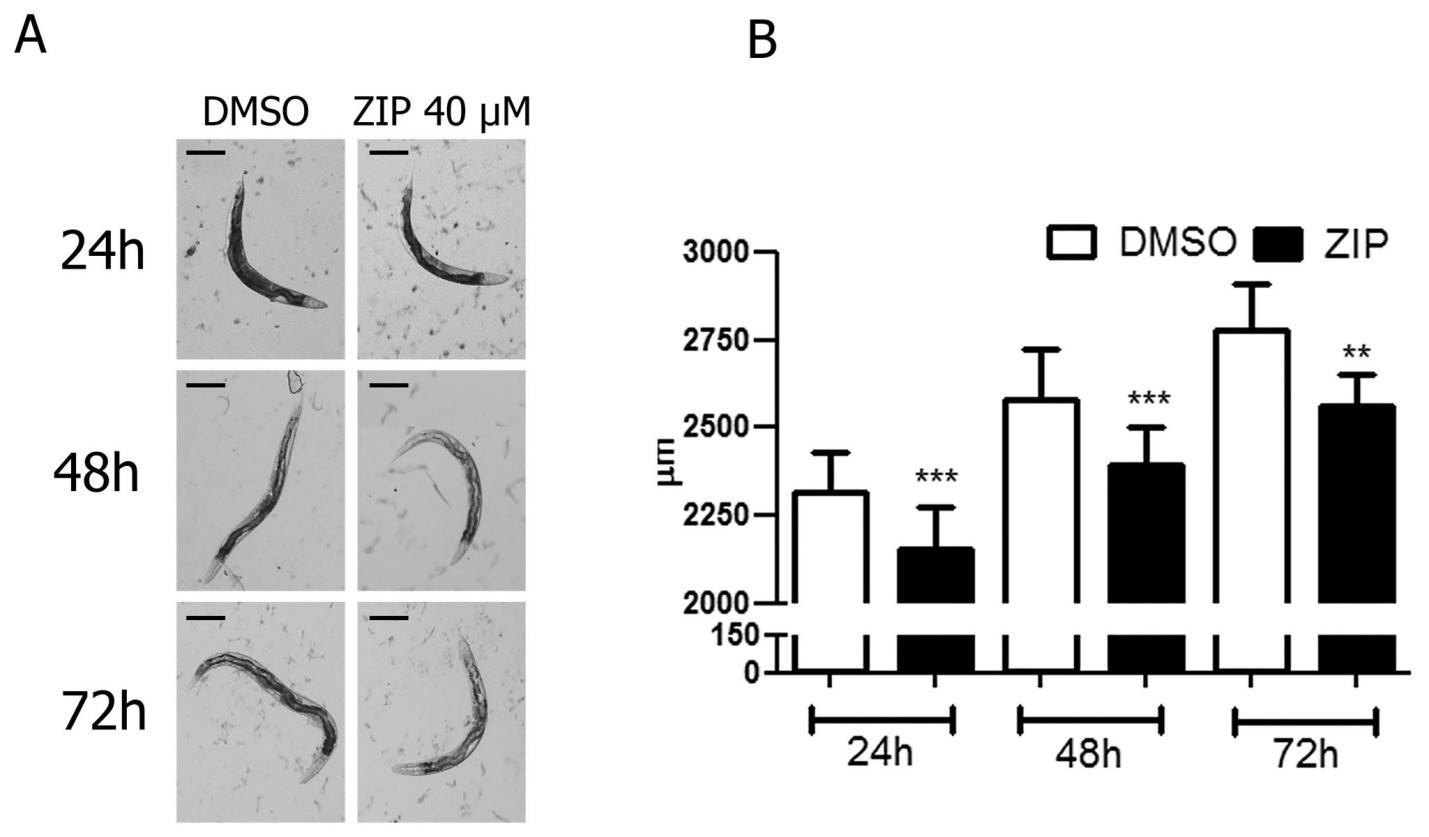
*Caenorhabditis elegans* wild-type (N2) perimeter after Ziprasidone treatment for 24, 48 and 72 hours. (A) Representative images of worms and (B) the perimeter quantification in µm after 24, 48 and 72 hours of Ziprasidone (40 µM) or vehicle control (DMSO 0.16% in Acetic acid (1:10 000)) exposition. After the determined times, the worms were removed of the plates and the images acquired with a stereoscope (40x, Nikon SMZ 1500 Stereoscope microscope). Statistically different compared to the respective control of each time (**p<0.001; ***p<0.0001, by Student’s T-test, mean, SD, n>20). The assays were confirmed twice.

**Figure 8:**
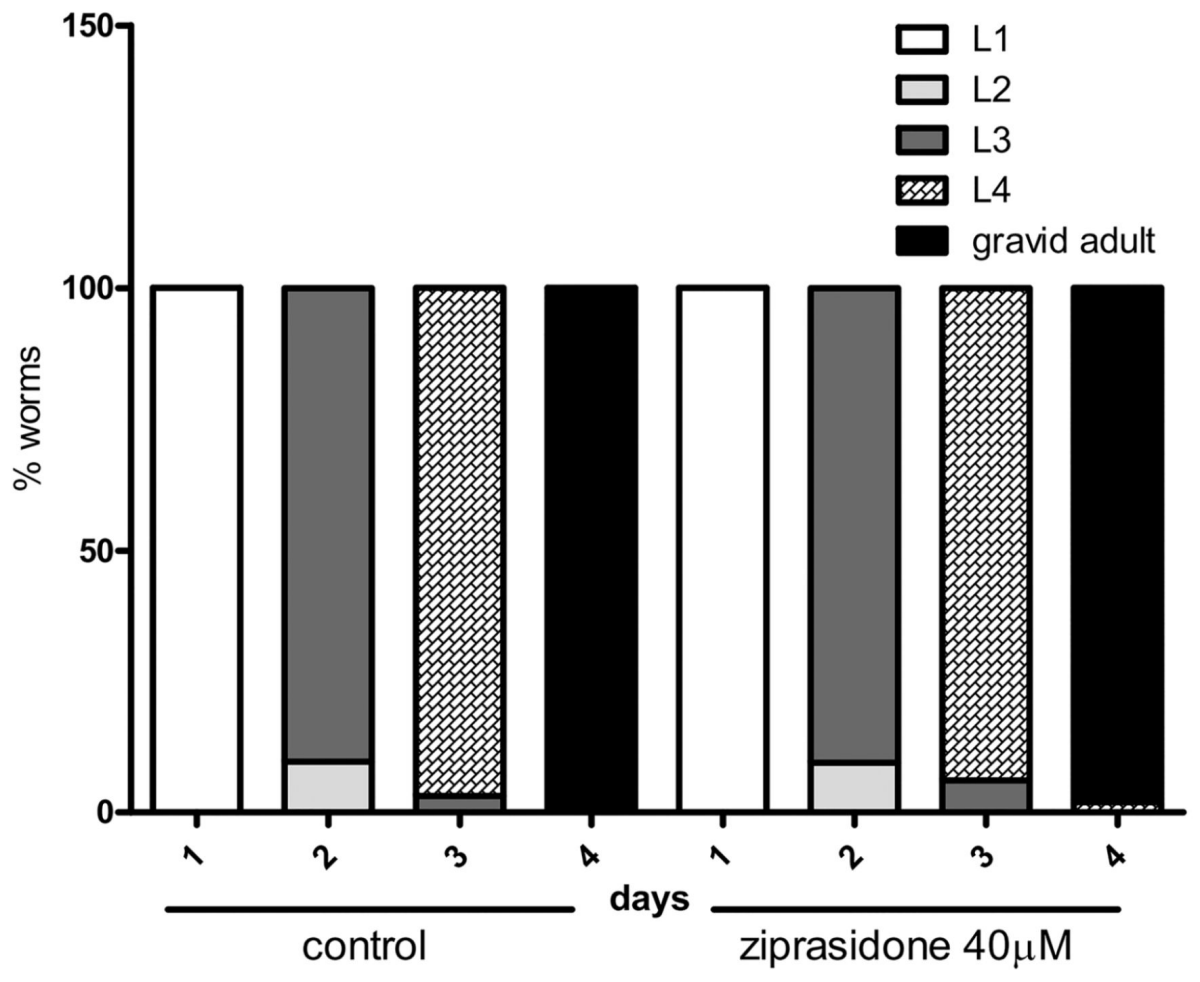
*C. elegans* larval development after Ziprasidone exposition. The worms were treated with Ziprasidone (40 µM) or control (DMSO 0.16% in Acetic acid (1:10 000)) after L1 larval stage until gravid adult. The results were evaluated by two way ANOVA no significant differences between groups were found (% worms in the larval stage, n=20).

Ziprasidone treatment also reduced body length (Figure 7) without altering larval development (Figure 8). Moreover, we did not detect any visual aberration in worm morphology. Combined, these data suggest that Ziprasidone may reduce fat depots by modifying mechanisms linked directly with fat regulation.

### Candidate genes related to Ziprasidone induced fat phenotype

**Figure 9:**
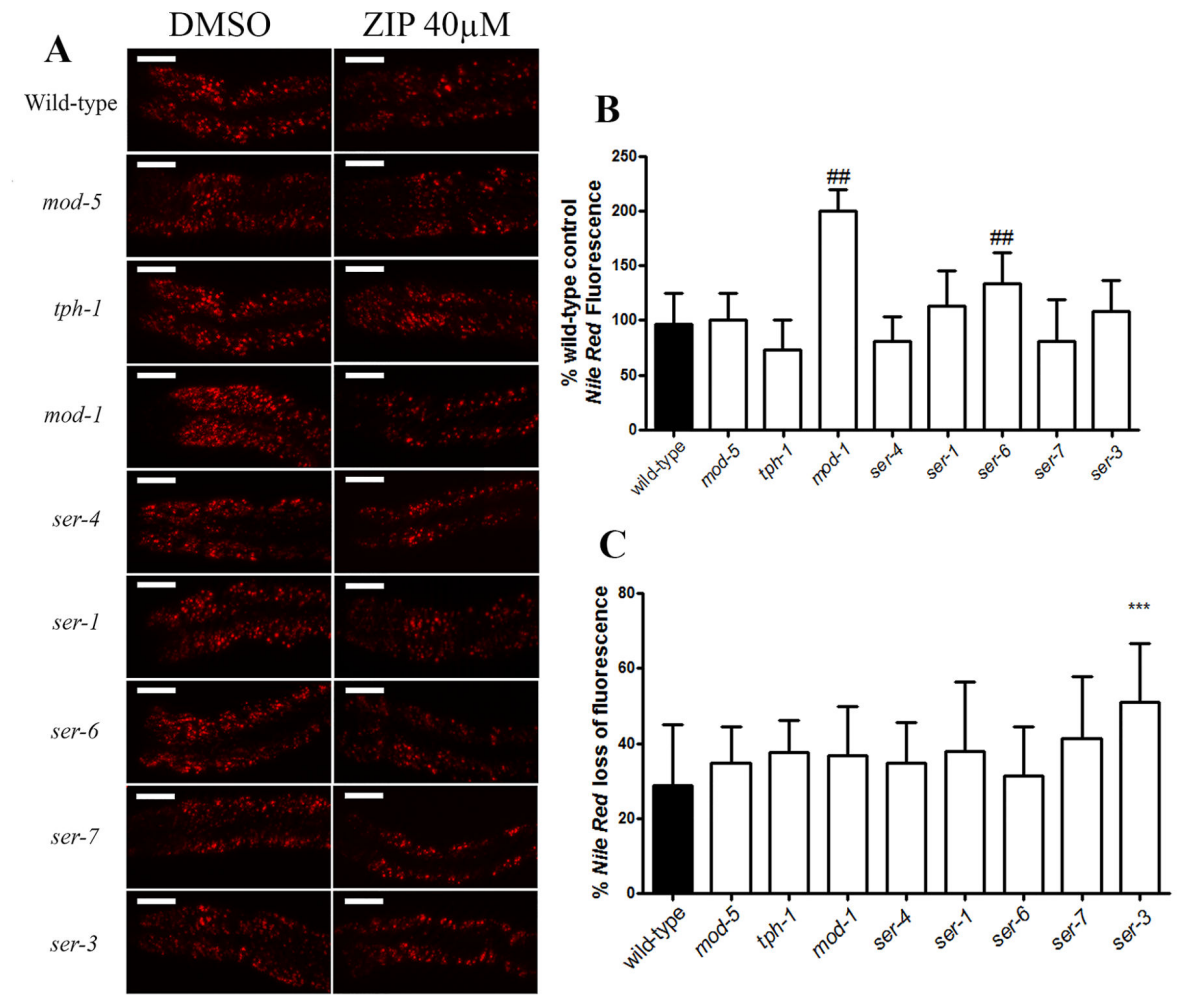
Ziprasidone effect on *Nile Red* fluorescence of *C. elegans* mutant to the serotoninergic. (A) Representative images of *Nile* *Red* fluorescence on *C. elegans* wild-type and mutants; (B) Fluorescence profile of *Nile* *Red* label on mutants exposed to the vehicle (0.16% DMSO in Acetic acid (1:10 000)) and (C) percentage of loss of *Nile* *Red* fluorescence on mutants exposed to Ziprasidone (40µM) compared to the wild-type worm (N2). The treatment began in the L4 larval stage until adult. ##p<0.0001 statistically different compared with wild-type worm exposed to the vehicle; ***p<0.0001 statistically different compared with wild-type worm under Ziprasidone (40µM) treatment (% of control, SD, n> 15, one way ANOVA with Bonferroni correction for *post* *hoc* multiple comparisons).

Serotonin receptors are among Ziprasidone’s pharmacological targets [18]. Studies have ascribed metabolic roles for serotonin [19]. In *C. elegans*, serotonin signaling pathways are linked to food intake and energy expenditure control [10]. To ascertain whether serotonin underlies the Ziprasidone-induced fat phenotype we assessed mutants carrying loss-of-function mutations in various components of the serotoninergic system (Figure 9). Of the components tested *mod-1* and *ser-6* had increased fat accumulation and all mutants reduced their fat stores in response to Ziprasidone treatment in a manner analogous to wild type, except for *ser-3*, which showed an enhanced response (Figure 9B). The *mod-1* fat phenotype was consistent with a previous study of fat control by serotonin in *C. elegans* [10]. Combined, these results suggest that *ser-3* aggravates the fat reduction induced by Ziprasidone (Figure 9C).

**Figure 10:**
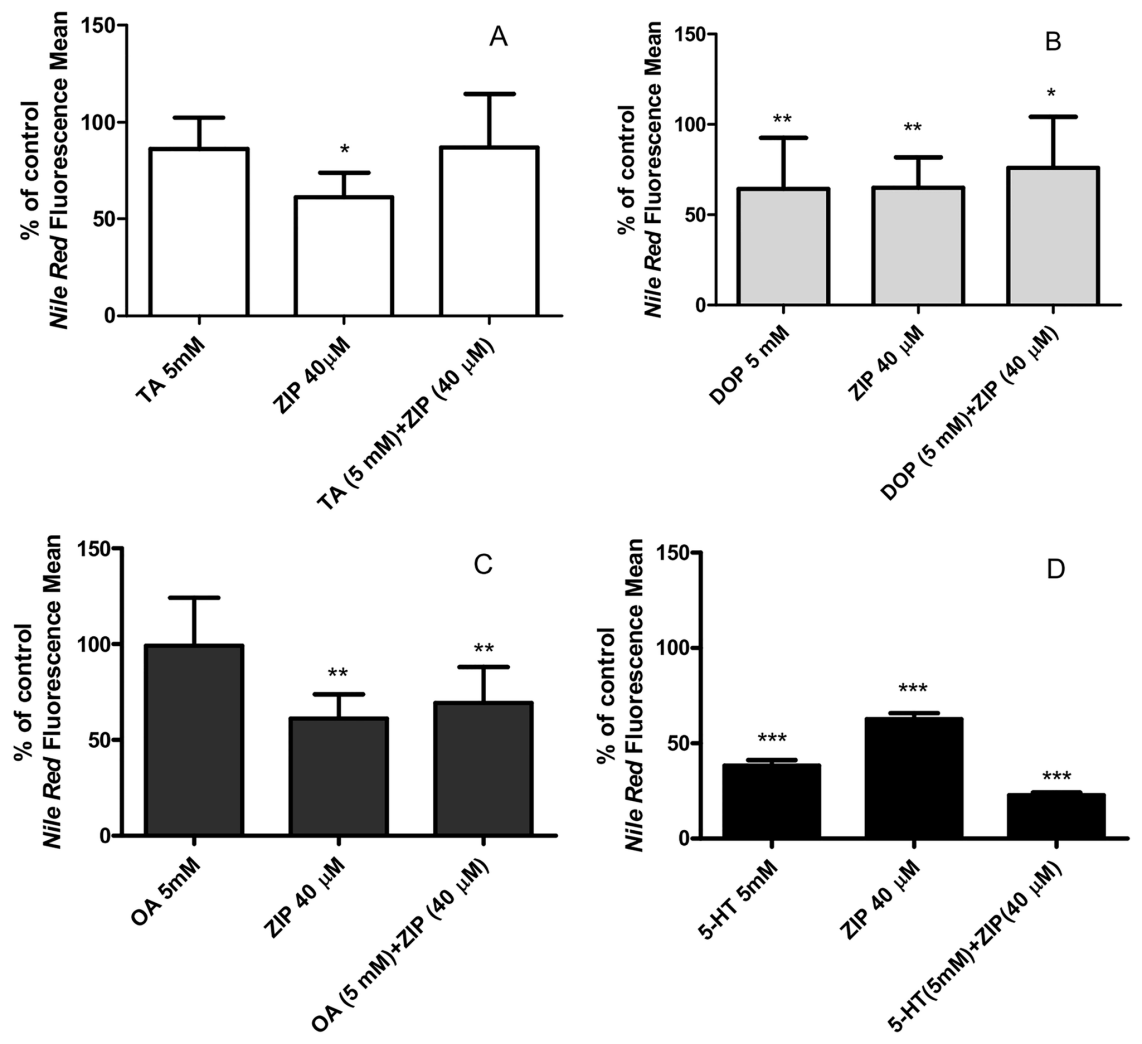
Effects on lipid stores after exogenous exposition to the monoamines and Ziprasidone in wild-type worms (N2). The nematodes were exposed to the monoamines (A) tyramine (TA, 5 mM), (B) dopamine (DA, 5 mM), (C) octopamine (AO, 5 mM) and (D) serotonin (5-HT, 5mM) isolated or concomitant to the Ziprasidone (40 µM). The fat amount was evaluated using *Nile* *Red* labeling follow density quantification in the first intestinal pair cells. The results were expressed in percentage of respective control: HCl 0.1N (compared to the monoamines), DMSO (0.16% in Acetic acid (1:10 000), compared to the Ziprasidone group) and HCl + DMSO (compared to the monoamine + Ziprasidone association). *p<0.05; **p<0.01; ***p<0.001, statistically different compared to the respective control by one way ANOVA with Bonferroni correction for *post* *hoc* multiple comparisons (% of control, SD, n=20).

Monoamines control numerous *C. elegans* behaviors associated with energy homeostasis. For instance, studies have shown that dopamine and octopamine may work antagonistically in signaling related to environmental food sources [20]. To determine whether Ziprasidone acts through other monoamine systems to cause fat reduction, we concomitantly treated the worms with exogenous neurotransmitters and antipsychotic. Of the monoamines tested, tyramine prevented fat reduction induced by Ziprasidone (Figure 10). This result suggests that the tyraminergic system may negatively regulate cellular signaling pathways targeted by Ziprasidone (Figure 10A).

**Figure 11:**
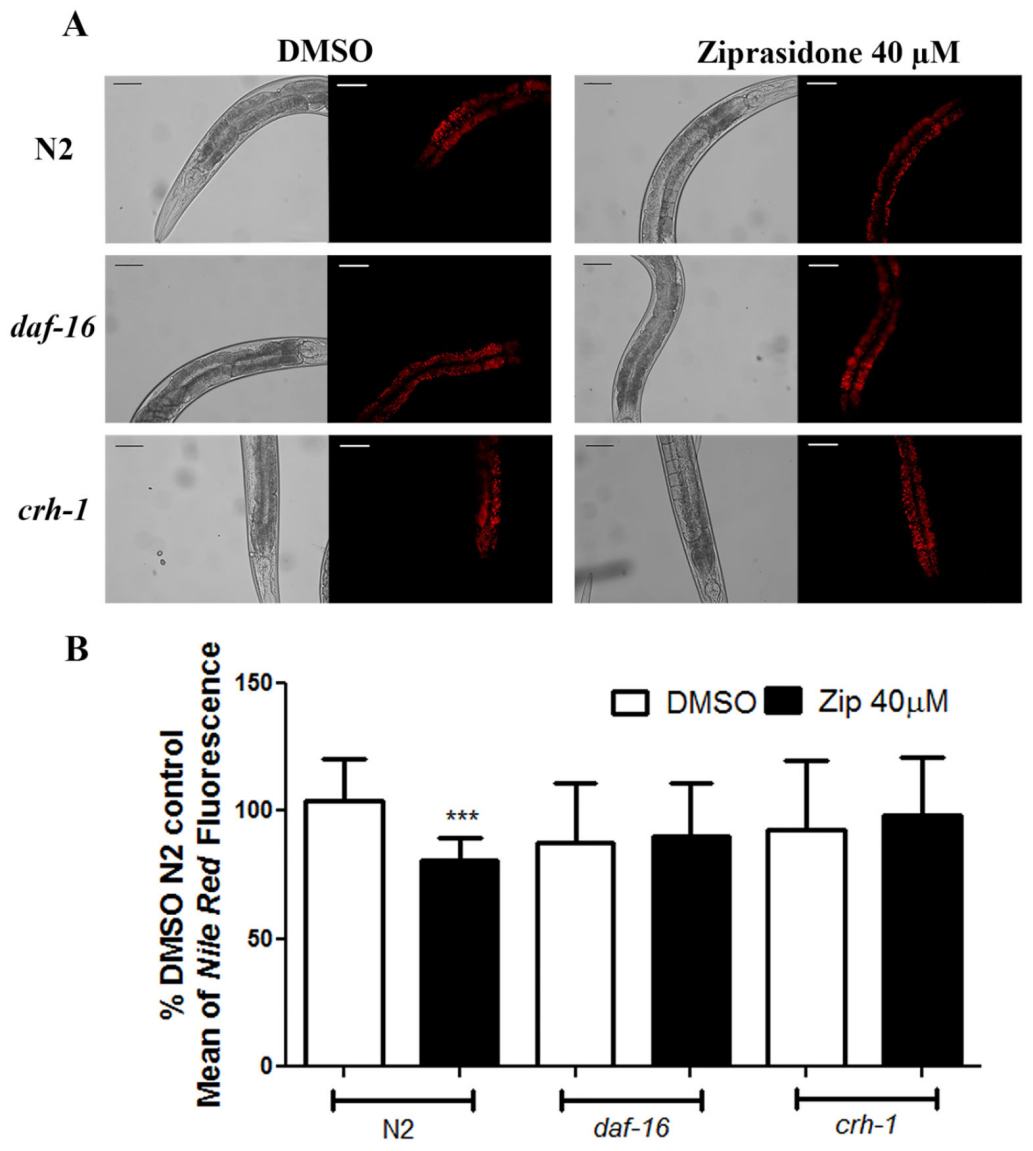
Recover of lipid profile through *Nile Red* labeling in mutants to *daf-16* and *crh-1* transcription factors after Ziprasidone treatment (40 µM). (A) Representative images of *Nile* *Red* fluorescence and (B) fluorescence amount in *C. elegans* mutants (*daf-16, crh-1*) and wild-type (N2). The results were expressed in percentage of control (DMSO 0.16% in Acetic acid (1:10 000)). ***p<0.0001, statistically different compared to the control by one way ANOVA with Bonferroni correction for *post* *hoc* multiple comparisons (mean of fluorescence, SD, n>25).

Octopamine signals starvation for activating *crh-1* [21], and both *crh-1* and *daf-16* are major regulators of fat metabolism [21,22]. To test whether Ziprasidone reduces fat by modulating these factors, we exposed loss-of-function *crh-1* and *daf-16* mutants to Ziprasidone and assessed the fat phenotype. Both mutations prevented Ziprasidone-induced reduction in fat depots (Figure 11). Our data are consistent with the ability of Ziprasidone to regulate fat metabolism through modulation of both *crh-1* and *daf-16*.

## Discussion

Ziprasidone is an atypical antipsychotic with fewer metabolic side effects compared to other members of this class. The molecular mechanisms underlying the unique metabolic profile of Ziprasidone are not clear. To identify related pathways, we used the nematode *C. elegans*. We found that Ziprasidone treatment reduced the worms’ fat stores. This was not an indirect outcome of modulation to other energy related behaviors and likely arises from direct interaction with neuroendocrine control of fat storage. Therefore, the phenotype observed mimics aspects of Ziprasidone’s metabolic profile in humans [5]. To shed light on components associated with Ziprasidone-induced fat phenotype, we exposed mutants carrying loss-of-function mutations in monoaminergic genes and transcription factors classically related with fat metabolism, and evaluated their responses. Of all mutants tested, we identified three that altered the wild type fat reduction triggered by Ziprasidone. *Ser-3* encodes for a putative serotonin, octopamine and tyramine receptor [21]. Our data demonstrate that loss of *ser-3* attenuates the Ziprasidone-induced fat reduction, since its absence enhanced the fat storage depletion elicited by the antipsychotic.

Fat storage regulation in *C. elegans* involves a complex network of transcription factors, and neuroendocrine signaling [23,24]. Serotonin, octopamine, tyramine and dopamine control behaviors involved in energy balance [25,8,26]. Concomitant treatment of monoamines and Ziprasidone, revealed that only tyramine was able to prevent fat storage reduction. It has been reported that tyramine signaling elicits increased food intake in *C. elegans*, a behavior normally induced by food deprivation [26]. Therefore, tyramine can directly or indirectly reverse the effects of Ziprasidone by modifying the food dynamic.

CRH-1 and DAF-16 are transcription factors with roles in fat metabolism modulation. CRH-1 can be activated by octopaminergic signaling on receptor SER-3 during starvation in *C. elegans* [21]. We observed that these factors abolished the effect of Ziprasidone on fat stores establishing their role in modulating fat deposition in worms.

Transcription factors function as molecular hubs for cell signaling pathways. Moreover, they are common downstream targets of monoamine-activated pathways such as the transcription factor cAMP response element-binding protein (CREB) [27,28,29]. *C. elegans* has a single CREB-ortholog gene, CRH-1. In the absence of food, CREB activation *in C. elegans* occurs in four cholinergic SIA neurons [30]. Starvation and disruption of insulin signaling are powerful signals in controlling energy metabolism, and CRH-1 and DAF-16 are key factors involved in this process [21,31].

The forkhead box O (FOXO) transcription factor DAF-16 factor is the major downstream regulator of insulin-like signaling. DAF-16/FOXO proteins are inactivated by the insulin/IGF-1 signaling (IIS) through PI3K and the AGC kinases Akt/SGK, which promote its cytosolic localization. Conversely, upon genetic alterations in IIS, several environmental factors, such as changes in temperature, oxidative stress and starvation, cause DAF-16 to translocate into the nucleus to promote target gene expression [32,33,34].

Antipsychotic drugs that induce weight gain, such as Clozapine were previously described as producing cytoplasmic localization of DAF-16::GFP in arrested L1 larvae. On the other hand, Clozapine treatment under starvation or high temperatures, produces nuclear localization of DAF-16::GFP in arrested L1 larvae [35]. Our data point that Ziprasidone only decreases the lipid accumulation in the presence of DAF-16 (Figure 11). It is possible that the opposite effect of Clozapine and Ziprasidone on DAF-16 localization explains their differential metabolic profiles. The results presented here suggest an involvement of both transcription factors in Ziprasidone’s effect on Nile Red fluorescence.

Fat stores reflect the balance between food intake and energy expenditure. We observed that Ziprasidone does not change the worms’ pharyngeal pumping rate, an indirect measure of food intake. Conversely, clozapine and olanzapine, two antipsychotics that produce correlative weight gain, decrease pharyngeal pumping rate [35]. It is noteworthy these studies used a higher concentration range compared to our study. Feeding behavior is a major determinant of fat deposit size and may partly explain the metabolic variability observed among antipsychotics. Ziprasidone treatment also resulted in a reduction in length absent any changes in larval developmental progression. This is in accordance with a previous report, where the same phenotype was observed with other antipsychotics [36]. Perhaps this is a trivial consequence of reduced energy stores although other worm mutants with reduced fat content are not always smaller.

Our results demonstrate both DAF-16 and CRH-1 transcription factors are necessary for the Ziprasidone-induced fat store reduction. It will be crucial in future studies to examine the effects of specific genes involved in lipid accumulation that may be downstream targets of DAF-16 and CRH-1, including lipases and lipid synthesis enzymes. *C. elegans* can be profitably used to uncover the molecular pathways linking Ziprasidone and lipid storage abnormalities, particularly due to the conservation of fat metabolism pathways between the worm and mammalian systems.
